# Targeting Autophagy in Atrial Fibrillation

**DOI:** 10.31083/j.rcm2410288

**Published:** 2023-10-08

**Authors:** Qiang Ye, Wen Chen, Hengsong Fu, Yanling Ding, Yuling Jing, Jingsong Shen, Ziyang Yuan, Kelan Zha

**Affiliations:** ^1^Department of Cardiology, The Affiliated Hospital of Southwest Medical University, 646000 Luzhou, Sichuan, China

**Keywords:** atrial anatomical remodeling, atrial electrical remodeling, atrial fibrillation, autophagy, energy metabolism remodeling

## Abstract

Atrial fibrillation (AF) is the most common type of arrhythmia in clinical practice, and its incidence is positively correlated with risk factors that include advanced age, hypertension, diabetes, and heart failure. Although our understanding of the mechanisms that govern the occurrence and persistence of AF has been increasing rapidly, the exact mechanism of AF is still not fully understood. Autophagy is an evolutionarily highly conserved and specific physiological process in cells that has been suggested as a potential therapeutic target for several cardiovascular diseases including the pathophysiology of AF. The present article provides an updated review of the fast-progressing field of research surrounding autophagy in AF, and how regulating autophagy might be a therapeutic target to reduce the incidence of AF.

## 1. Introduction

Current research shows that the prevalence of atrial fibrillation (AF) in adults is 2–4%, and AF is quickly becoming the most common form of tachyarrhythmia worldwide [1]. Great progress in understanding the pathophysiology of AF has occurred over the past 20 years. Arrhythmogenic remodeling is any change in structure or function that leads to arrhythmia and is central to most acquired atrial fibrillation [2]. Atrial remodeling includes atrial electrical remodeling (AER) and atrial anatomical remodeling (AAR). AER indicates a shorter atrial action potential duration (APD), a shorter atrial effective refractory period (AERP), and a slower atrial conduction velocity [3]. AAR, particularly atrial fibrosis, is important in the progress of many types of atrial fibrillation. The persistence of atrial fibrillation is caused by fibrosis, therefore the development of fibrosis is used as both a potential therapeutic target and a predictor of treatment response [2].

Although the ultimate mechanism of AF is still unknown, there are many hypotheses, such as the multiple-wavelet hypothesis, the focal impulse hypothesis, atrial remodeling, and the neurohumoral regulation mechanism. However, no effective drugs corresponded to these hypotheses. The demand to find new therapeutic targets for AF has intensified [3]. Autophagy, an elaborate and ubiquitous mechanism, is involved in the turnover of organelles and proteins. Autophagy is essential for cell survival, differentiation, development, and homeostasis. Thus far, a potential link between AF and changes in autophagic activity has gained increasing attention [4]. Autophagy is involved in atrial AER, AAR, energy metabolism remodeling, and neural remodeling. It plays different roles in atrial remodeling under different cellular micro-environmental conditions. Differential gene expression analysis, functional enrichment analysis, and protein-protein interaction analysis of autophagy in AF have suggested that autophagy-related genes (ARG) have potential biomarkers and therapeutic targets in AF [5]. Recently, a comprehensive analysis of ARG and valvular AF has indicated that ARG may be potential predictive markers and therapeutic targets for determining a treatment strategy for patients with AF [6]. Whether autophagy is a marker of failing cardiomyocyte repair or a self-cleaning pathway for failing cardiomyocytes, remains unclear. Autophagy protects the cardiac structure and function from stress injury under baseline conditions, limiting damages under most of conditions [7]. Thus, this paper will review the recent research on the link between cardiac autophagy and AF, and the potential role of targeted autophagy in the treatment and prevention of AF.

## 2. Autophagy

### 2.1 Autophagic Pathways

**Fig. 1.**
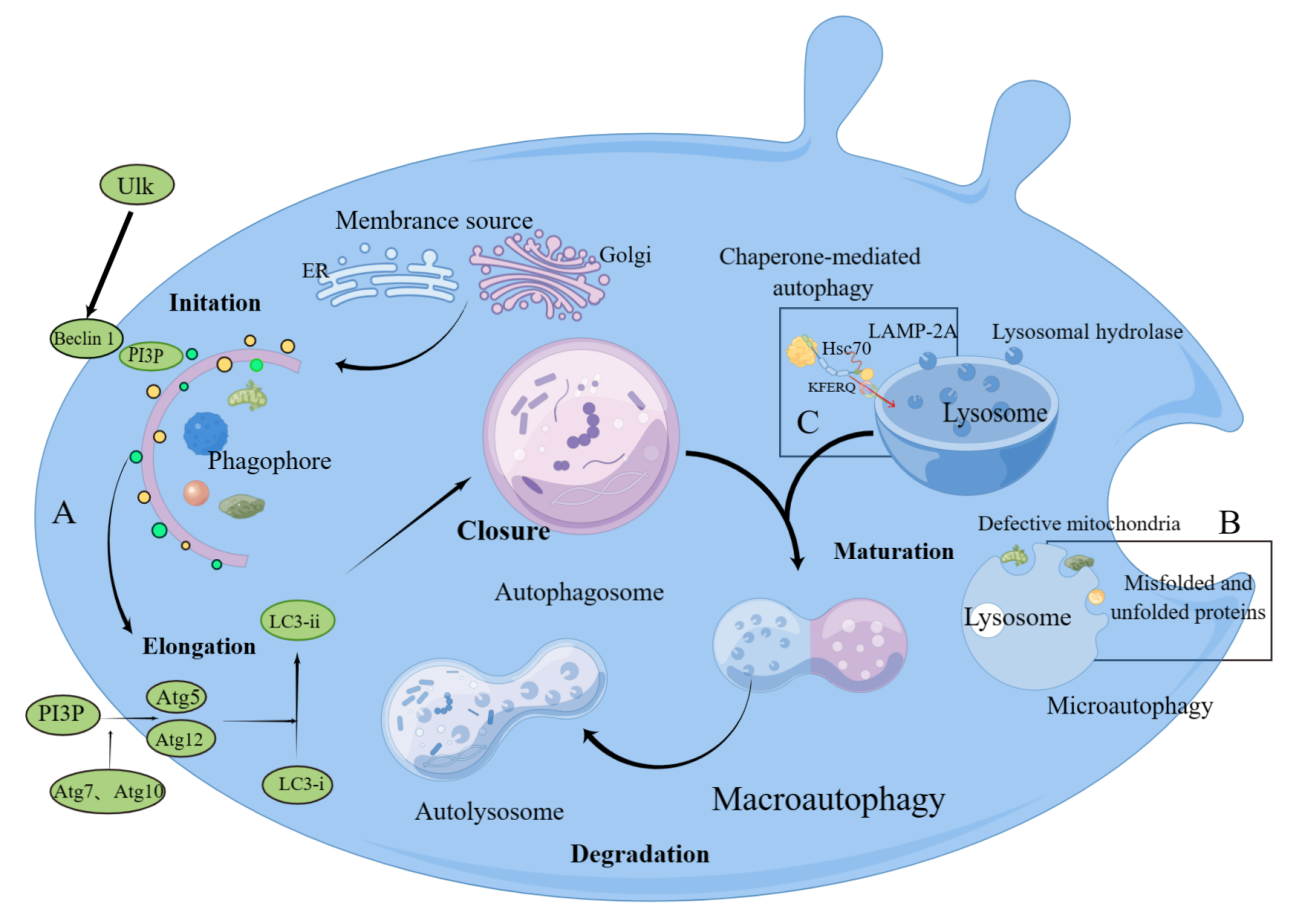
**Schematic model of the major pathways of the autophagic mechanisms.** (A) Macroautophagy of intracellular substances includes initiation, elongation, closure, maturation, and degradation. The endoplasmic reticulum, Golgi apparatus, mitochondria, and plasma membranes are membrane sources of autophagic vesicles. The Ulk macromolecular complex promotes the initiation of the phagophore, thereby regulating PI3P formation to control nascent autophagosomes. Varieties of autophagy-related proteins are involved in this process. The autophagosome sequesters proteins, both misfolded and unfolded, and defective mitochondria. After maturation, autophagosomes fuse with lysosomes to form the autolysosome where the contents are degraded by acid hydrolases. (B) In microautophagy, lysosomes directly engulf cellular components. (C) In CMA, Hsc70 transports the target protein to the lysosome for degradation. ULK (UNC51-like) enzymes are a family of mammalian kinases that have critical roles in autophagy and development. PI3P, phosphatidyl inositol triphosphate; Atg, autophagy related gene; LC3, microtuble-associated protein light chain 3; ER, endoplasmic reticulum; Hsc, heat-shock cognate protein; LAMP, lysosome-associated membrane glycoprotein; CMA, chaperone-mediated autophagy; Hsc70, heat shock cognate 70.

Depending on the route by which the autophagy substrate is delivered to the lysosomal body, autophagy can be divided into three types: macroautophagy, microautophagy, and chaperone-mediated autophagy (Fig. 1). Macroautophagy (hereafter referred to simply as autophagy) is the formation of autophagosomes that wrap cellular proteins, debris, and organelles and deliver them to lysosomes for degradation (Fig. 1A) [8]. Microautophagy occurs when peptides, small proteins, and other small molecules recognized by cells as potentially toxic are removed, which involves the direct uptake of target substances by lysosomes for recycling (Fig. 1B) [9]. The process by which heat shock cognate 70 (Hsc70) selectively binds exposed KFERQ-based proteins and pushes them into the lysosome via the LAMP-2A (lysosome-associated membrane protein type 2A) receptor is known as chaperone-mediated autophagy (CMA) (Fig. 1C) [9]. Macroautophagy has been extensively studied in heart diseases. It removes damaged and superfluous organelles and proteins to reestablish cellular homeostasis [10]. Macroautophagy is mediated by ubiquitin and enzyme-containing complexes ULK (unc-51–like autophagy-activating kinase) and PI3P (phosphatidylinositol 3-phosphate). The assembly of Atg (autophagy-related gene) 12-Atg5 complexes is mediated by PI3P, which in turn stimulate the conversion of LC3 (microtubule-associated protein 1 light chain 3) I to LC3 II. Many ARGs are involved in the occurrence of autophagy. Beclin1 protein and LC3 are used as biomarkers of the autophagy process. Beclin1 and LC3 promote the formation and elongation of phagophore membrane respectively. In the past decade, significant progress has been made in the study of the molecular mechanisms, regulatory mechanisms, and pathophysiological effects of autophagy. Much research has suggested that mutations in ARGs are associated with various cardiovascular diseases [11].

### 2.2 Autophagy and Risk Factors of Atrial Fibrillation

Around the world, atrial fibrillation is the most common arrhythmia in adults. Aging is considered the main risk factor for AF, but other comorbidities, such as hypertension, diabetes and inflammation, also increase the risk for AF [1].

#### 2.2.1 Diabetes

Diabetes causes oxidative stress and contractile dysfunction in the heart. Previous studies have confirmed that autophagy is inhibited by diabetes, which in turn increases endoplasmic reticulum stress and phosphorylation of pro-hypertrophic signaling molecules [12]. Advanced glycation end products (AGE) play a role in diabetic cardiac dysfunction. Autophagy is inhibited by knocking out the AGE receptor, endothelial-to-mesenchymal transition is suppressed, and thereby cardiac fibrosis are attenuated [13]. Defective autophagy and NLRP3 (NOD-like receptor pyrin domain-containing-3) inflammatory vesicle activation caused by diabetes mellitus, cardiac remodeling after myocardial infarction exacerbated by excessive secretion of pro-inflammatory cytokines [14]. Impaired autophagy is associated with cardiac metabolic dysregulation and oxidative stress in diabetic patients and is an important cause of cell death, fibrosis, and cardiac dysfunction [15].

#### 2.2.2 Age

Autophagy has been shown to be involved in the mechanisms of occurrence in a range of age-related diseases, including cardiovascular disease. Autophagic activity declines with aging, which may be one of the causes of age-related cardiac dysfunction [16]. Autophagy is involved in the organismal lifespan; impaired autophagy is associated with age-related mitochondrial dysfunction and the resulting accumulation of reactive oxygen species (ROS) [17]. Autophagy has been shown to be an important component of intracellular homeostasis, limiting ROS production by degrading dysfunctional mitochondria [18]. Impaired mitochondrial autophagic clearance has multiple consequences, including increased risk of arrhythmia, reducing contractile reserve, and increasing inflammation; age-related dysregulation of autophagy is central to the link between autophagy, ROS, and aging [16].

#### 2.2.3 Inflammation

Inflammation is associated with many mechanisms related to the development of multiple diseases, including cardiovascular disease. Autophagy has been shown to regulate the degree of inflammation. Autophagy is accompanied by activation of inflammatory vesicles and moderates inflammation by eliminating active inflammatory vesicles [19]. Stimulation of interferon gene over-expression may mitigate cardiac inflammation by pressure overload through inhibition of autophagy [20]. Interventions, including the use of autophagy inhibitors, can lead to higher interleukin-1β (IL-1β) levels [21]. Autophagy and inflammation are interdependent processes.

### 2.3 Autophagy Regulation

Both excessive activation and inhibition of autophagy can lead to cell death. Therefore, the regulation of autophagic balance is critical to the treatment of cardiac remodeling. Autophagy has been shown to be involved in all cardiomyopathies [21]. FYCO1 (the novel FYVM and coiled-coil domain-containing protein) is an important protein that regulates autophagy and is essential for adaptation to cardiac stress. FYCO1 interacts directly with LC3, Rab7 (a late endosome-/lysosome-associated small guanosine triphosphatase), and phosphatidylinositol-3-phosphate to promote end-directed transport of microtubules and autophagic vesicles [22]. Significant inhibition of autophagic flux after starvation was observed in cardiomyocytes with reduced or absent levels of FYCO1 and in FYCO1-deficient mice [23]. Ghrelin is an intestinal hormone that has been shown to be protective against cardiac dysfunction and remodeling. Lu’s study [24] indicated that ghrelin may exert cardioprotective effects during myocardial hypertrophy by promoting autophagy, possibly through the ${\mathrm{Ca}}^{2+}$/calmodulin-dependent protein kinase kinase (CaMKK)/adenosine $5^{\prime }$-monophosphate-activated protein kinase (AMPK) signaling pathway. It is becoming clear that altered autophagic activity is associated with cardiovascular diseases.

## 3. The Role of Autophagy in AF

Just as aging is an important risk factor for AF, other conditions that promote atrial remodeling, such as hypertension, diabetes, obesity, heart failure, and coronary artery disease, are also common [1]. Atrial fibrosis, electrical conduction abnormalities, and apoptosis play important roles in AF-promoting pathologies. Autophagy can cut both ways; moderate autophagy activation improves the survival rate of cells in response to adverse stress, whereas autophagy activation caused by excessive stimulation can lead to an apoptotic process called type II programmed death, which features excessive autophagic degradation [25]. Constitutive autophagy is beneficial for cell survival, and stress-induced autophagy plays both beneficial and detrimental roles in cardiomyocyte function and survival [26]. In an ischemia/reperfusion injury model, cardiomyocyte autophagy is increased in response to stress, yet impaired cellular autophagosome clearance during stress is associated with cell death [27]. In patients with postoperative AF, microtuble-associated protein light chain 3 (LC3) BⅡ is markedly reduced, suggesting impairment in the autophagic flow [28]. LC3B, a subtype of LC3, can also be used as a marker of autophagy. The LC3B processing refers to the transformation process of LC3BⅠ to LC3BⅡ. In a study in which canine atrial tissue was stimulated with high-frequency electric current, thereby establishing AF, abnormal activation of autophagy was detected in the canine atrial myocytes. The same result was obtained in atrial myocytes of patients with AF, suggesting that the imbalance of autophagy may be a potential pathogenic factor of AF [29].

### 3.1 Atrial Electrical Remodeling

#### 3.1.1 Connexin Remodeling

**Fig. 2.**
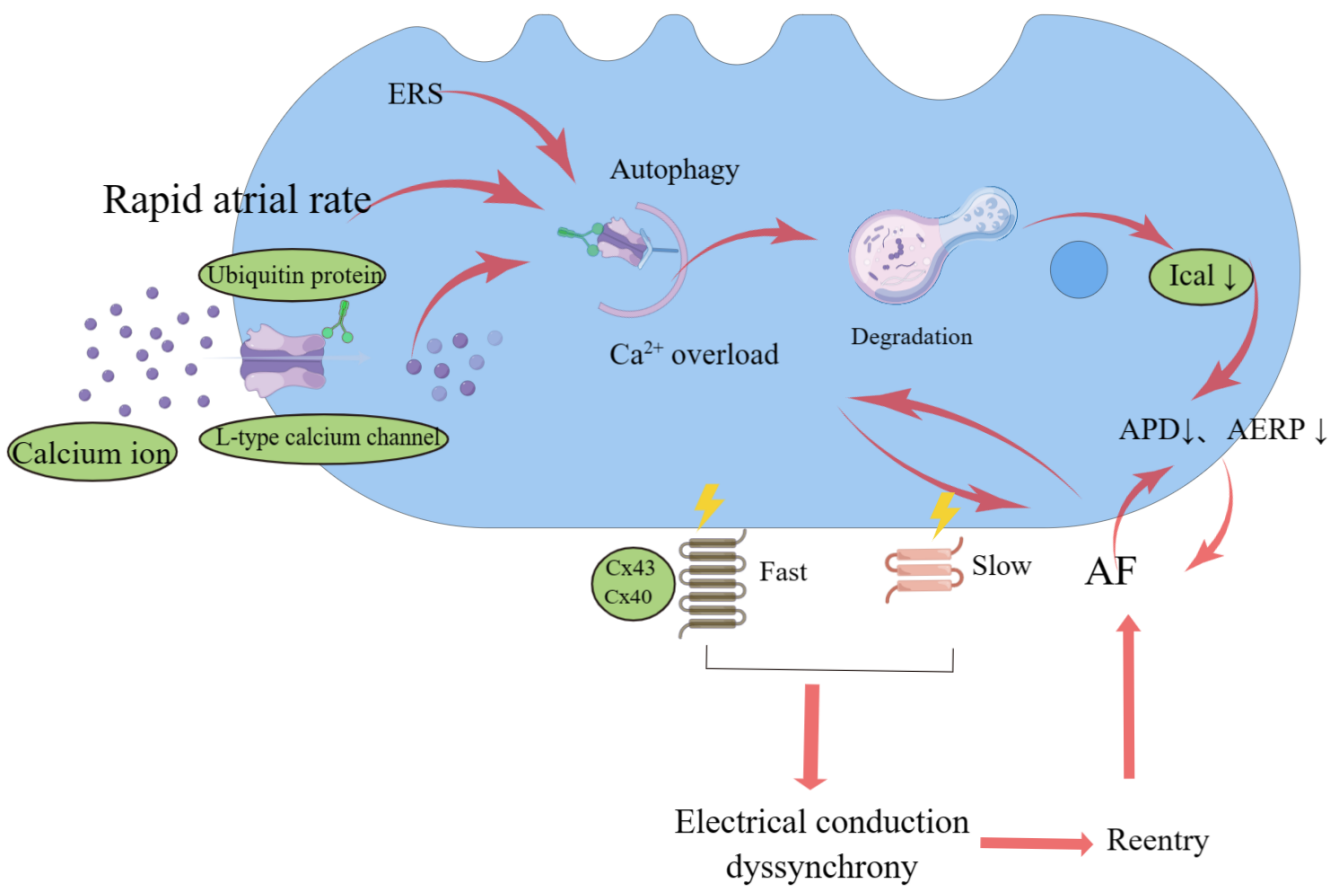
**Schematic diagram of autophagy involved in atrial electrical remodeling (AER).** Rapid atrial rates increase the influx of ${\mathrm{Ca}}^{2+}$ into atrial cells at each action potential, leading to calcium overload, and then inducing atrial fibrillation (AF). AF can also lead to calcium overload. Activated autophagy induced by rapid atrial rates and ERS induces a decrease in action potential duration (APD) and a shortened atrial effective refractory period (AERP) via ubiquitin-dependent selective degradation of Ical. Shortened APD and atrial fibrillation reinforce each other in a vicious cycle. Activated autophagy promotes connexin degradation, leading to different impedances and conduction velocities between cardiomyocytes, resulting in micro-reentrant and inducing AF. ERS, endoplasmic reticulum stress; Ical, L-type calcium channel; Cx, connexin.

Gap junctions and ion channels form a pathway for electrical conduction between cardiomyocytes (Fig. 2). Changes in the structure, function, and characteristics of the atrial ion channels are thought to be pivotal mechanisms of AER [3]. Connexin (Cx) forms gap junctions and is responsible for the propagation of cardiac action potentials between cardiomyocytes [4]. Cx40 and Cx43 are the most important members of the connexin family. Reduced numbers or abnormal distribution of Cx43 may result in different impedances and conduction velocities between cardiomyocytes, in turn resulting in micro-reentrant and AF susceptibility [30]. Connexins have an unexpectedly short half-life of only 1–5 h, raising the question of how cardiomyocytes regulate connexin degradation so quickly. In recent years, several studies have demonstrated that autophagy is involved in connexin degradation. Several studies on different tissues, primary cultured cells, and cell lines have shown that gap junctions are influenced by constitutive and induced autophagy. Ultrastructural analyses have revealed cytoplasmic annular gap junctions inside phagophores, indicating that autophagy is a connexin-degradation pathway [31]. Chen *et al*. [32] found that the mammalian target of rapamycin (mTOR) inhibitor rapamycin promoted autophagy, further inhibiting the protective effect of apelin-13 on Cx43. Their finding demonstrated that increased autophagy inhibited cardiac Cx43 expression [32]. Thus, regulation of autophagic flux to preserve Cx43 remodeling may represent a new strategy for AF management.

#### 3.1.2 Calcium Channel Remodeling

Cardiac depolarization activates voltage-dependent calcium channels on the myocardial membrane and calcium ions enter the cell, inducing ryanodine receptor 2 (RyR2) opening on the sarcoplasmic reticulum, thereby triggering a calcium transient. In the AF model of the right atrium in fast-pacing dogs, the openness of the RyR2 receptor was significantly increased in the atrial-fibrillation group. Therefore, the increased openness of the RyR2 receptor is speculated to lead to the leakage of sarcoplasmic reticulum ${\mathrm{Ca}}^{2+}$ into the cytoplasm during diastole, thereby causing AF [33]. Autophagy markers and the key Atg7 of cardiomyocytes are significantly increased in patients with AF. Inhibition of autophagy by lentivirus-mediated Atg7 knockdown, or the autophagy inhibitor chloroquine (CQ), restores the shortened AERP and alleviates the AF vulnerability caused by tachypacing in rabbits [34]. High-frequency electrical stimulation of fast-pacing atria also activates autophagy of cardiomyocytes and promotes autophagy-mediated degradation of the L-type calcium channel protein by ubiquitin protein, further shortening the APD and AERP. These processes make AF more likely to occur and persist [34]. Endoplasmic reticulum stress (ERS)-associated autophagy can induce AER, which appears as L-type calcium channel reduction [35]. By fluorescent immunostaining, we found a significant increase in the expression of the autophagy marker LC3 in the left atrial tissue of rats after myocardial infarction, and a significant reduction in protein expression of the L-type calcium channel α1c, voltage-gated sodium channel 1.5, and voltage-activated A-type potassium ion channel 4.3; however, this was completely reversed by the small-molecule integrated stress-response inhibitor [36].

### 3.2 The Effect of Autophagy on Myocardial Fibrosis

Myocardial fibrosis is a pathological process that causes extracellular matrix (ECM) accumulation in the myocardium, which can cause cardiac systolic and diastolic dysfunction resulting in AF. Myocardial fibrosis is one of the main factors leading to cardiac structural remodeling and AF. ECM is also involved in atrial structural remodeling. Many researchers have proposed that atrial fibrosis may be a potential key factor and biomarker for the pathogenesis of AF [37, 38]. Therefore, the prevention or improvement of atrial fibrosis is crucial for the prevention of AF and for the restoration of cardiac function. Studies have increasingly shown that impaired or hyperactive autophagy plays an important role in the occurrence of myocardial fibrosis and AF.

**Fig. 3.**
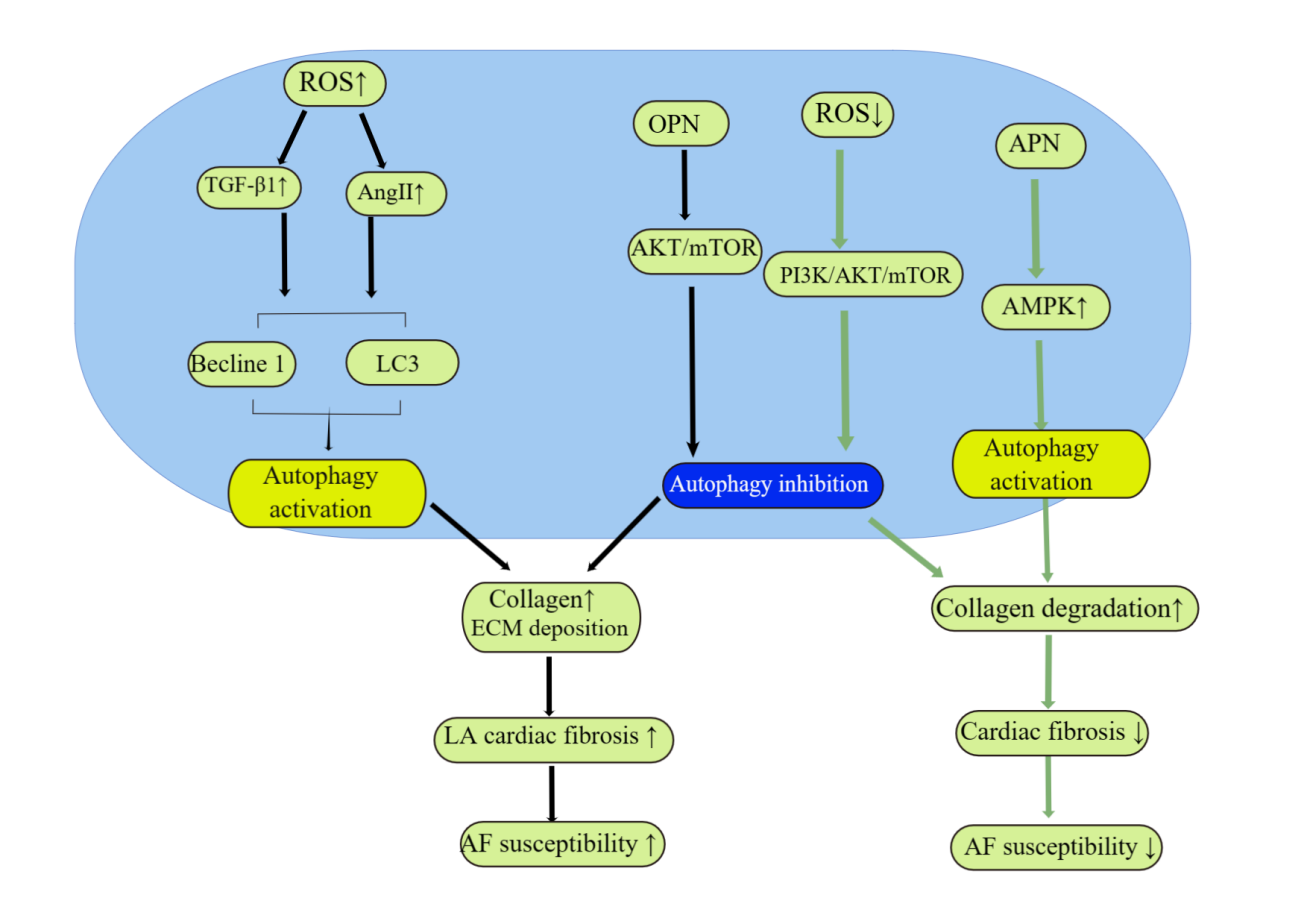
**Schematic diagram of autophagy involved in atrial anatomical remodeling (AAR).** Autophagy may exert a dual role in regulating the extracellular matrix in myocardial fibrosis. Myocardial fibrosis can impede electric propagation, favoring reentry. Either autophagy activation or inhibition may promote atrial fibrillation. ROS, reactive oxygen species; Ang II, angiotensin II; TGF-β1, transforming growth factor-β1; LC3, microtuble-associated protein light chain 3; ECM, extracellular matrix; OPN, osteopontin; AMPK, adenosine $5^{\prime }$-monophosphate (AMP)-activated protein kinase; APN, adiponectin; mTOR, mammalian target of rapamycin; PI3K, phosphatidylinositol 3-kinase; AKT, protein kinase B; LA, left atrial; AF, atrial fibrillation.

Myocardial fibrosis causes inhomogeneity of electrical conduction between myocardial bundles, which may induce unevenness of current conduction, shortening of functional potentials, depolarization of resting cardiomyocytes, and induction of spontaneous phase 4 depolarization, when atrial ectopic electrical signals encounter this vulnerable matrix [39]. The cardiac fibroblast-to-myofibroblast transition has been shown to contribute to cardiac fibrosis. Atrial fibrosis leads to AF, whereas atrial rapid pacing also leads to the accumulation of extracellular matrix, forming a vicious circle of “fibrosis-AF-fibrosis”. Although autophagy is involved in several diseases, little is known about the role of autophagy in cardiac fibrosis. Autophagy plays different, even opposite, roles in different specific states of the disease or its stress factors (Fig. 3). Whether autophagy is beneficial or detrimental to cardiac fibrosis, especially atrial fibrosis and atrial fibrillation, is still controversial.

#### 3.2.1 Activated Autophagy Exacerbates Myocardial Fibrosis and Induces AF

Angiotensin II (Ang II) and transforming growth factor-β1 (TGF-β1) are important molecules for atrial fibrotic remodeling in AF [37]. Ang II is a major effector of the renin-angiotensin system and plays an important role in the regulation of collagen deposition and interstitial fibrosis. Ang II increases collagen secretion from atrial fibroblasts; enhanced fibroblast autophagy is also observed. When the autophagy inhibitors 3-methyladenine or chloroquine (CQ) were used to block autophagy, collagen secretion was significantly reduced, suggesting that activated autophagy may promote myocardial fibrosis [40]. Homoplastically, primary human atrial myofibroblasts were treated with TGF-β1 to assess fibrogenic and autophagic responses. The results showed that TGF-β1 led to an elevation of autophagic markers and fibronectin, and pharmacological inhibition of autophagy decreased the fibrotic response, these results support that TGFβ1-induced autophagy increases the fibrogenetic response [41]. Inflammatory processes lead to renin-angiotensin-aldosterone system activation and electrical and structural remodeling. Previous studies have shown that this inflammatory condition is often accompanied by releasing of ROS, which is closely related to the development and progression of cardiac fibrosis [42]. A recent study demonstrated that excessive ROS increases autophagosome formation by inhibiting the phosphatidylinositol 3-kinase (PI3K)/protein kinase B (AKT)/mTOR signaling pathway, and eventually enhanced cardiomyocyte apoptosis [43]. Therefore, autophagy may be considered as a potential mechanism for the formation of atrial fibrosis.

#### 3.2.2 Autophagy Activation Attenuates Myocardial Fibrosis

Excessive extracellular matrix deposition in the cardiac interstitium is characteristic of cardiac fibrosis. Inhibition of myocardial fibrosis is a potential approach to prevent and treat AF. Increasingly, studies have shown that promoting autophagy can inhibit myocardial fibrosis and improve cardiac function. Ang II has been established as an effective inducer of extracellular matrix protein. Autophagy also promotes excessive collagen degradation, which attenuates Ang II-induced cardiac fibrosis [44]. Adiponectin (APN), also known as adipocyte complement-related protein, has been shown to possess anti-inflammatory properties and to attenuate myocardial fibrosis. The inhibitory effect of APN on cardiac inflammation and fibrosis may be achieved by enhancing autophagy in macrophages [45]. In addition, in AF model cells and rat myocardial tissues, isoprenaline induces myocardial fibrosis, whereas quercetin-activated autophagy reverses this process [46].

Previous studies have shown that autophagy is critical for the regulation of extracellular matrix homeostasis. Autophagy is activated by many cellular signaling factors such as oxidative stress, volume overload, and inflammation, and it directly degrades collagen and fibronectin. The autophagic degradation of collagen and fibronectin induces adverse cardiac remodeling [47]. Overactive adrenergic stimulation has been demonstrated to stimulate fibroblast autophagic procollagen digestion, which contributes to reduced fibrosis by regulating the extracellular matrix [48]. Autophagy-associated collagen degradation is a compensatory mechanism for myocardial fibrosis. In contrast, autophagy suppresses cardiac fibrosis by regulating the secretion of pro-fibrotic factors. TGF-β1 stimulates fibroblast growth and extracellular matrix production, resulting in dysfunction of atrial tissues. The secretion of TGF-β1 strictly depends upon secretory autophagosome intermediates, in fibroblasts and macrophages, as carriers. Notably, all treatments interfering with autophagosome formation inhibited TGF-β1 release [49]. Osteopontin (OPN), which contributes to various tissue fibrosis processes, is highly expressed in the circulation of patients with AF. It promotes atrial fibrosis *in vitro*, and further increases with the progression of AF. *In vitro* studies in human atrial fibroblasts demonstrated that OPN activates AKT, a suppressor of autophagy, which suppresses autophagy and increases atrial collagen I and fibronectin production. However, this process was reversed by rapamycin, an autophagy inducer [50].

### 3.3 Atrial Energy Metabolic Remodeling

In 2004, van Bilsen proposed the concept of myocardial energy metabolism remodeling, including changes in myocardial substrate utilization, mitochondrial dysfunction, and reduced cardiac high-energy phosphate [51]. A recent study showed that knockout of heat shock protein 22 impaired cardiac autophagy, disrupted cardiac energy metabolism, and increased oxidative damage [52]. The rapid contraction of the atria in AF inevitably leads to a surge in the energy demand. Autophagy is known to participate in the pathophysiological process [53]. An increasing number of studies have shown that autophagy is involved in protein, lipid, and glucose metabolic remodeling of cardiomyocytes in AF.

#### 3.3.1 Autophagy and Protein Metabolism in AF

**Fig. 4.**
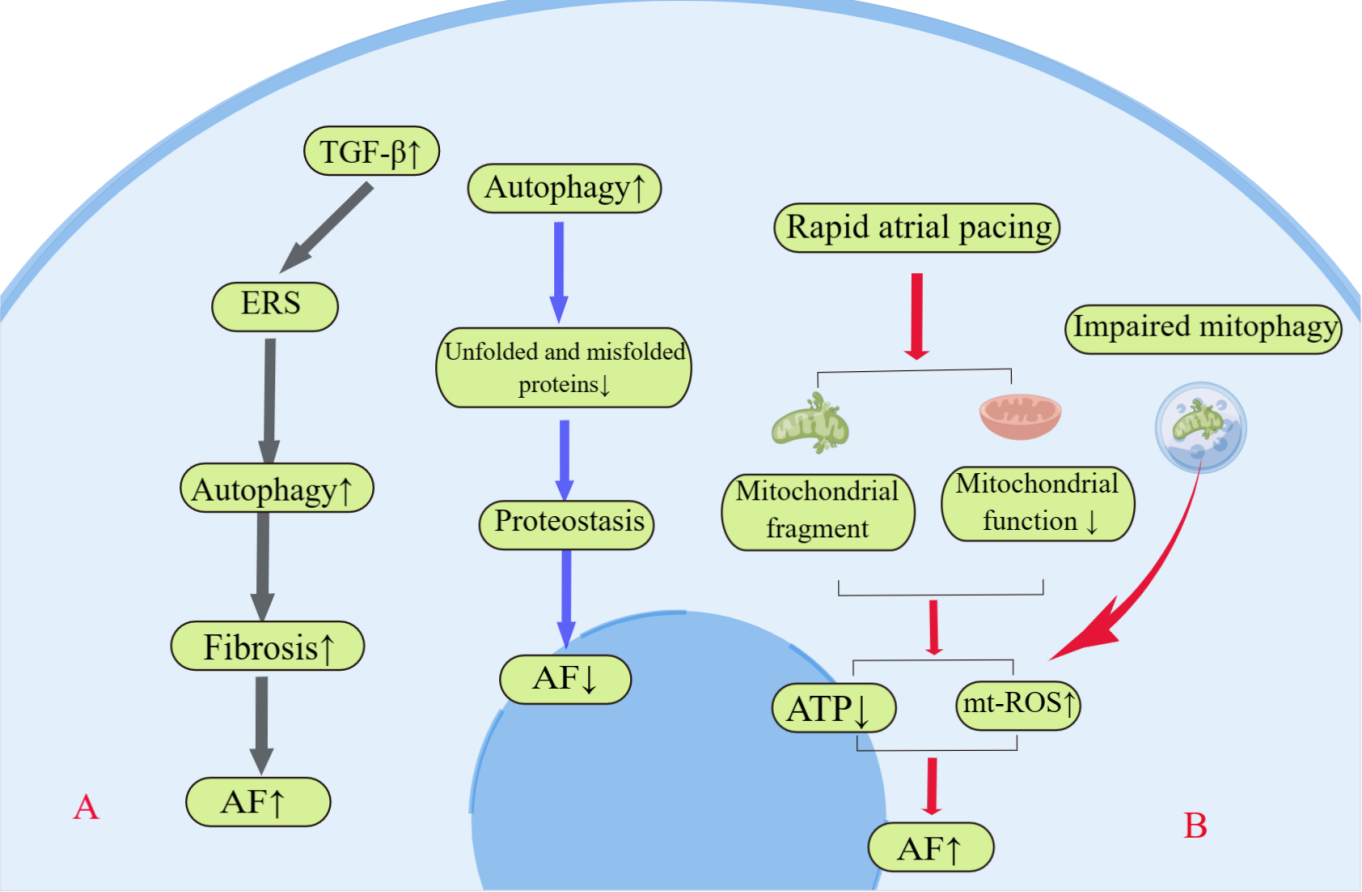
**Schematic diagram of autophagy involved in proteostasis and mitochondrial function.** (A) Autophagy protects cardiomyocytes from atrial fibrillation (AF) by degrading unfolded and misfolded proteins. But there are other views that ERS-induced autophagy also increases AF susceptibility by aggravating myocardial fibrosis. (B) Rapid atrial pacing leads to mitochondrial fragmentation and impaired function, which in turn leads to increased mt-ROS and decreased ATP. Impaired mitophagy causes a decrease in dysfunctional mitochondria as the potential mechanisms. mt-ROS, mitochondrial reactive oxygen species; TGF-β1, transforming growth factor-β1; ATP, adenosine-triphosphate; ERS, endoplasmic reticulum stress.

Proper protein folding is a key mechanism for maintaining cell and tissue function; the strict regulation of the production and folding of these proteins is known as proteostasis. Cells have a precise cellular protein quantity control (PQC) network. However, if the protein is unable to fold correctly, the body will digest and degrade the proteins through the ubiquitin-proteasome system or autophagosome-lysosome pathway, which prevents downstream dysfunction [54]. Autophagy maintains cellular proteostasis by regulating PQC. On the one hand, autophagy mitigates the accumulation of misfolded proteins by degrading potentially toxic molecules and organelles in cardiomyocytes. On the other hand, autophagy acts as a cellular recycling program to recycle amino acids, lipids, and other molecular building blocks liberated from substrates with the help of lysosomal acid hydrolases [55]. The critical role of proteostasis in AF has been extensively demonstrated in numerous studies [56]. Endoplasmic reticulum stress is a protective stress response to the accumulation of misfolded and unfolded proteins and has been observed to increase autophagy and to promote atrial remodeling in animal models and in the cardiac tissue of patients with AF (Fig. 4A). *In vivo* treatment with the chemical chaperone 4-phenyl butyrate, an inhibitor of ERS, prevents autophagy activation, protects atrial-tachypacing canine cardiomyocytes from electrical remodeling, and attenuates AF progression [35]. In a H9c2 cardiomyoblast model of ERS, TGF-β activates ERS autophagy and cell injury, whereas an autophagy inhibitor protects H9c2 cardiomyoblasts from myocardial fibrosis by attenuating ERS autophagy [57]. Notably, similar results were obtained after treatment with another autophagy inhibitor. Tetrameric DsRed (a red fluorescent protein) causes proteinopathy and cellular injuries, and it has been shown to promote severe fibrosis. In transgenic mice expressing tetrameric DsRed, the ubiquitin-proteasome system and autophagy-lysosome systems were impaired and overburdened [58]. The derivation of proteostasis leading to atrial remodeling is an important cause of the self-sustained characteristics of AF, and autophagy may be the pivotal mechanism for maintaining protein homeostasis.

Atrial structural remodeling is rooted in derailment of the homeostasis of production, function, and breakdown of proteins, including degradation of structural and contractile proteins. Decreased levels of acetylated α-microtubulin in atrial tissue of patients with paroxysmal and persistent atrial fibrillation coincide with increased histone deacetylase-6 (HDAC6) expression and activity [59]. Restoration of the microtubule network is the key to recovery of structural and functional reconstruction after rapid pacing [60]. HDACs modulate atrial cardiomyocytes ${\mathrm{Ca}}^{2+}$ signaling and contractility by regulating protein acetylation status of α-tubulin. Activated HDAC6 was observed in tachypacing HL-1 atrial cardiomyocytes, and the same transformation was observed in atrial tissue from patients with AF. Fast pacing induces HDAC6-specific activation leading to α-microtubulin deacetylation, depolymerization, and degradation [61]. McLendon *et al*. [62] showed that hyperacetylated tubulin is an adaptive response that can enhance autophagy in the face of proteotoxicity. In addition, Song *et al*. [63] also found that ɑ-tubulin hyperacetylation protected cardiomyocytes against doxorubicin-induced acute damage by recovering impaired autophagy flux. AF induces remodeling partly through HADC6 activation; derailment of ɑ-tubulin proteostasis may be a potential mechanisms and therapeutic target for AF.

#### 3.3.2 Autophagy and Lipid Metabolism in AF

Fatty acid oxidation is the main energy source of cardiomyocytes, and elevated levels of serum-free fatty acids are strong risk factors and outcome predictors of AF and AF-related stroke [64]. Increased expression of genes related to atrial fatty acid metabolism in patients with AF positively correlates with atrial structural- and electrical-remodeling levels [65]. Thus, abnormalities in atrial fatty acid metabolism are involved in structural and electrical remodeling of the atria, which may contribute to the future development or recurrence of atrial fibrillation. Autophagic vesicles selectively degrade excess intracellular lipids to maintain the balance of fatty acid metabolism; this is called “lipophagy” [66]. Autophagy prevents the accumulation of toxic fatty acid metabolites (e.g., diacylglycerols) in cells by degrading intracellular lipids [65]. Lipophagy, a unique form of selective autophagy, is a fundamental mechanisms of lipid clearance [53]. Previous research has shown that high-fat intake aggravates cardiac remodeling, including hypertrophy and interstitial fibrosis. Mitochondrial aldehyde dehydrogenase (ALDH2) serves an indispensable role in protecting against cardiac anomalies from high-fat-induced obesity by enhancing autophagy [67]. Modifying autophagy-mediated protein clearance is a potential new strategy for regulating cardiac lipoprotein lipase levels, which regulate fatty acid levels in the heart [68]. Dysfunction of energy metabolism in the atrial tissue may be one of the pathogenic mechanisms of AF.

#### 3.3.3 Glycometabolism and Autophagy in AF

The relationship between diabetes mellitus and AF is well established. Enhanced p62 levels and decreased levels of autophagy-related gene 7 (ATG7) were observed in cardiac cells treated with high levels of glucose, indicating impaired autophagy, whereas ALDH2 alleviated diabetes-induced cardiac mechanical and geometric changes, which are mediated by the regulation of autophagy [69]. Significant deposition of collagen fibers, significant increase of senescent cells in myocardial tissue, significant inhibition of cellular autophagy, increased aging of myocardial cells, and increased fibrosis in diabetic myocardium were observed in myocardial tissue of diabetic rats [69]. Autophagy is activated in diabetic rats during cardiac fibrosis, and autophagic activity is correlated with cardiac fibrosis [70]. In a rat model of type 2 diabetes, trimetazidine therapy enhanced cardiac autophagy and reduced myocardial fibrosis and cardiomyocyte apoptosis [71].

#### 3.3.4 Mitophagy in AF

The source of adenosine-triphosphate (ATP) in cardiac tissue is critically dependent on mitochondrial oxidative phosphorylation. Mitochondria are organelles of cardiomyocytes associated with energy metabolism. Accumulation of dysfunctional mitochondria significantly increases the production of mitochondrial reactive oxygen species (mt-ROS), initiating the endogenous cell death process and resulting in myocardial cell damage. Mitochondrial dysfunction leads to reduced ATP production, metabolic dysregulation, increased ROS production, and the activation of apoptotic cascades, which are associated with the development of arrhythmogenic atrial electroanatomical remodeling [72]. Increasing evidence suggests that mitochondrial dysfunction is associated with AF. Rapid atrial pacing created an animal model of AF, and AERP, AF induction rate, and mitochondrial DNA were determined [73]. Reduced mitochondrial DNA content and downregulation of mitochondrial respirators were observed in rapidly paced atria, demonstrating a dysfunction of mitochondrial energy metabolism [73]. Atrial biopsies from patients with AF also exhibited aberrant ATP levels, upregulation of mitochondrial stress chaperones, and fragmentation of the mitochondrial network [74]. Montaigne *et al*. [75] found that patients with impaired mitochondrial function in the atrial myocardium had a higher incidence of AF after coronary artery bypass graft surgery, indicating that damaged mitochondrial accumulation may be a potential factor for the occurrence of AF.

Selective autophagic removal of mitochondria (mitophagy) is a crucial homeostatic mechanism that promotes proper functioning of the mitochondrial network. The elimination of dysfunctional mitochondria through mitophagy could contribute to maintaining normal function and the number of mitochondria [72]. Mitochondrial quality and content are properly regulated by mitochondrial autophagy during cell differentiation. Inhibition of B-cell lymphoma-2 (BCL2) interacting protein 3 like (BNIP3L)- and FUN14 domain containing 1 (FUNDC1)-mediated mitochondrial division during differentiation leads to sustained mitochondrial fission and donut-shaped impaired mitochondria [76]. However, both excessive reduction and increase in mitochondrial clearance contribute in part to cardiovascular dysfunction. Mitophagy is involved in metabolic remodeling of several cardiovascular diseases, such as cardiac hypertrophy, myocardial infarction, ischemia/reperfusion, heart failure, hypertension, and diabetic cardiomyopathy [77]. Mitochondrial autophagy is essential for maintaining the balance between mitochondrial degradation and accumulation. Increasing evidence has revealed that mitochondrial dysfunction provides an arrhythmogenic substrate for the development and perpetuation of AF [57, 74, 75]. Based on these findings, it is reasonable to suppose that impaired mitophagy may contribute to AF (Fig. 4B). Patients with chronic AF with defective cardiac autophagy and an increased number of dysfunctional mitochondria provided convincing evidence that impaired mitophagy is a potential mechanism of human chronic AF [5].

## 4. Strategies and Challenges

Autophagic fluxes are involved in cardiovascular physiology and pathophysiology in myriad ways, in essentially all cell types. An Food and Drug Administration (FDA)-approved histone deacetylase (HDAC) inhibitor, suberoylanilide hydroxamic acid (SAHA, Vorinostat), reduces myocardial infarction size in a large animal model by reactivating ischemia/reperfusion-triggered downregulation of autophagy [78]. However, there are still some challenges that need to be addressed. As mentioned previously, the role of autophagic flux in cardiovascular pathophysiology may be bidirectional. It is important to effectively target these strategies to specific cells and tissues, because globally altered autophagic flux may be beneficial in one tissue and detrimental in other parts of the body [21].

## 5. Conclusions

Atrial fibrillation is the most common clinical arrhythmia. It is characterized by a series of significant changes in the electrical, structural, and functional properties of the atria. AF is the result of multiple factors, and due to the irreversibility of atrial remodeling and the limited effect of drug therapy and catheter ablation, it brings a serious burden to the lives of patients with AF. Autophagy is an evolutionarily conserved physiological process that degrades abnormal proteins, toxic metabolites, and damaged organelles, thereby protecting the myocardium. However, abnormally activated autophagy that promotes atrial remodeling has also been widely reported. In recent years, accumulating evidence has shown that autophagy may contribute to AF, the possible mechanisms including atrial fibrosis, electrophysiological remodeling, and energy metabolic remodeling. The relationship between autophagy and AF has been described in detail. Increasing data obtained from animal and cell models have demonstrated that modulation of autophagy may be a promising approach for the treatment of AF. Although great advances have been made in cardiac autophagy research over the past decade, further research is needed in order to apply this approach in clinical practice. Therefore, new research methods to assess autophagy in human subjects should be developed.
